# Inhibition of PI3K/Akt Pathway Impairs G2/M Transition of Cell Cycle in Late Developing Progenitors of the Avian Embryo Retina

**DOI:** 10.1371/journal.pone.0053517

**Published:** 2013-01-02

**Authors:** Isis Moraes Ornelas, Thayane Martins Silva, Lucianne Fragel-Madeira, Ana Lucia Marques Ventura

**Affiliations:** Department of Neurobiology, Neuroscience Program, Institute of Biology, Fluminense Federal University, Niterói, Rio de Janeiro, Brazil; Seattle Children's Research Institute, United States of America

## Abstract

PI3K/Akt is an important pathway implicated in the proliferation and survival of cells in the CNS. Here we investigated the participation of the PI3K/Akt signal pathway in cell cycle of developing retinal progenitors. Immunofluorescence assays performed in cultures of chick embryo retinal cells and intact tissues revealed the presence of phosphorylated Akt and 4E-BP1 in cells with typical mitotic profiles. Blockade of PI3K activity with the chemical inhibitor LY 294002 (LY) in retinal explants blocked the progression of proliferating cells through G2/M transition, indicated by an expressive increase in the number of cells labeled for phosphorylated histone H3 in the ventricular margin of the retina. No significant level of cell death could be detected at this region. Retinal explants treated with LY for 24 h also showed a significant decrease in the expression of phospho-Akt, phospho-GSK-3 and the hyperphosphorylated form of 4E-BP1. Although no change in the expression of cyclin B1 was detected, a significant decrease in CDK1 expression was noticed after 24 h of LY treatment both in retinal explants and monolayer cultures. Our results suggest that PI3K/Akt is an active pathway during proliferation of retinal progenitors and its activity appears to be required for proper CDK1 expression levels and mitosis progression of these cells.

## Introduction

The PI3K/Akt pathway is a signal transduction pathway activated by several membrane receptors such as growth factor and G protein-coupled receptors. Activated PI3K converts the lipid phosphatidylinositol 4,5 biphosphate (PtdIns (4,5)P2) to phosphatidylinositol 3,4,5 triphosphate (PtdIns (3,4,5)P3) that recruits Akt to plasma membrane where it is activated by phosphorylation at two sites. 3′-phosphoinositide-dependent kinase-1 (PDK1) phosphorylates Thr308 residue at Akt activation loop [1], whereas mTORC2 complex phosphorylates Ser473 residue situated at the hydrophobic site [2]. Akt catalyzes phosphorylation of several protein targets like GSK-3, BAD, p27kip1 and tuberin. PI3K/Akt pathway regulates diverse cellular functions such as cell survival, energy metabolism, cellular motility and cell cycle progression [3] and deregulation of its activity contributes to cell transformation and diabetes [4], [5].

Cell cycle progression is promoted by the activity of phase-specific kinase complexes composed of cyclins and cyclin-dependent kinases (CDKs) and a role for PI3K/Akt pathway in G1/S transition was suggested. Akt prevents cyclin D1 degradation by regulating the activity of GSK3 [6] and can negatively influence the expression of the CDK-inhibitor p27/kip1 through direct phosphorylation of its Thr157 residue that promotes the cytoplasmic retention of this protein [7], [8], [9].

Onset of mitosis is controlled by activation of Cyclin B1/ CDK1 complex, the activity of CDK1 being highly regulated [10]. This enzyme, that is inactive as a monomer, must bind cyclin B during late G2 when it is phosphorylated at Thr161 by CDK-activating kinase that allows Cyclin B/CDK1 complex activation [11]. In addition, when bound to cyclin B during interphase, the complex is held in an inactive state by phosphorylation of CDK1 at Thr14 and Tyr15 residues catalyzed by the protein kinases Myt1 that phosphorylates both residues and Wee1 that phosphorylates Tyr15. At the G2/M transition, phosphorylated and inhibited CDK1 is dephosphorylated by Cdc25 phosphatase, which leads to an abrupt activation of Cyclin B/CDK1 complex.

PI3K/Akt pathway has been shown to be involved in meiotic maturation in several species. In primary oocytes, cell cycle arrest generally occurs at the first prophase of meiosis and the release of these cells is induced by several hormones. In Xenopus, mouse, starfish and porcine oocytes, resumption of meiosis can be interrupted by PI3K or Akt inhibition that keeps maturation promoting factor (MPF or cyclin B/CDK1 complex) inactive [12], [13], [14], [15], [16], [17]. In these cells, Akt directly phosphorylates Myt1 at Ser75 and inactivates it, reversing the balance between Myt1 and Cdc25 activities, thus initiating the activation of cyclin B–CDK1 to cause meiotic G2/M phase transition [15].

Some previous evidences suggest a role of PI3K in G2 and M phases of the cell cycle in somatic cells. In synchronized cell lines, Akt is active during mitosis and inhibition of PI3K/Akt promotes a delay in S phase exit and G2/M transition due a decrease in CDK1 activity [18], [19]. However, still limited information is available regarding the mechanisms activated by PI3K in the control of G2 and M phases especially in the control of cell cycle of neuronal and glial progenitors.

The neural retina is a widely used model for approaching the development of the CNS [20] and the genesis of all retinal cell types during development is now well characterized. The spatio-temporal development of the various cell types is also well characterized in the retina. Within the early stages of development, mitosis is confined to retinal progenitors located at the proliferative ventricular zone of the retina known as the neuroblastic layer (NBL) immediately adjacent to retinal pigmented epithelium [21]. Cell cycle in the proliferative zone of the retina is tightly controlled and proceeds in synchrony with interkinetic migration of neuroblast nuclei along the NBL. Cell nuclei occupy different positions along the retinal axis and mitosis occurs at the outer, apical margin of the NBL while DNA synthesis occurs at the inner, basal margin [22]. This spatial segregation of cells along the cell cycle phases and the flexibility of isolating and culturing retinal cells facilitate experimental studies of the cell cycle in the retina.

An important regulation of cell proliferation mediated by activation of nucleotide receptors was observed in this tissue where activation of P2Y2/4 and P2Y1 receptors induces the proliferation of early and late developing progenitors, respectively [21], [23], [24], [25], [26], [27]. In late developing progenitors of the chick embryo retina, nucleotide-induced cell proliferation involves activation of PKC, MAP kinases and the PI3K/Akt pathway that mediates an increase in cyclin D1 expression in these retinal progenitors [28]. Moreover, the involvement of PI3K in retinal cell proliferation was also demonstrated in transgenic mice expressing a mutated PI3K (p65^PI3K^) that is constitutively active. In the retina of these animals, increased cell proliferation at early stages of development was observed [29].

In the present work, we investigated the role of PI3K/Akt pathway in cell cycle of retinal progenitors. Our data revealed that Akt is active during mitosis and inhibition of PI3K/Akt pathway promotes cell cycle arrest at G2/M transition by regulating CDK1 expression rather than cyclin B1 expression.

## Experimental Procedures

All procedures were performed according to the Guidelines for the Care and Use of Laboratory Animals, as described in the ARVO Statement for the Use of Animals in Ophthalmic and Vision Research, and by the National Institute of Health, and approved by the commission of animal care CEPA/PROPPi from Federal Fluminense University.

### Materials

Fertilized White Leghorn chicken eggs were obtained from a local hatchery and incubated at 38°C in a humidified atmosphere up to the appropriate stage. LY294002 (PI3K inhibitor) and Anti-BrdU antibody (B2531) were from Sigma-Aldrich (St. Louis, MO, USA); Minimum Essential Medium (MEM), Fetal Calf Serum were from Life Technologies (São Paulo, SP, Brazil). Antibodies against phospho-Akt (Ser473, (catalog # 4060), Akt (catalog # 4691), phospho-4E-BP1 (Thr37/46, catalog # 2855), phospho-Histone H3 (Ser10, catalog # 3377 and # 9706), phospho-GSK-3 (Ser21/9, catalog # 9331), cyclin B1 (catalog # 4138), phospho-CDK1 (Tyr15, catalog # 9111), CDK1 (catalog # 9112), PCNA (catalog # 2586), ERK (catalog # 9102), cleaved caspase-3 (catalog # 9664) were from Cell Signaling Technology (MA, USA). Anti-phospho-Akt antibody (Ser473, catalog sc-7985) from Santa Cruz Biotechnology, anti-α-tubulin (catalog T5168) from Sigma Aldrich, anti- α-transitin (EAP3) from DSHB and anti-γ-tubulin (catalog # 11316) from Abcam were also used. All other reagents were of analytical grade.

### Retinal preparations

Both monolayer cultures and intact retinas from 8-day-old embryos were used to perform immunolabeling for pAkt, pH3 and p4E-BP1. When PI3K inhibition was performed with LY294002, intact tissues were replaced by retinal explants in the immunofluorescence experiments. Protein extracts of both monolayer cultures and explants were used for western blotting of proteins when PI3K was inhibited.

### Retinal cell monolayer cultures

Chick embryos at embryonic day 7 (E7) were killed instantaneously by decapitation and the eyes were removed and immediately transferred to Ca^2+^- and Mg^2+^-free balanced salt solution (CMF) where the retinas were dissected from other structures. Trypsin, at a final concentration of 0.1%, was then added to the tissues and the suspension incubated at 37°C for 20–25 min. Trypsin solution was removed and the retinas suspended in MEM containing 2% fetal calf serum, 2 mM glutamine, 100 U/ml penicillin and 100 µg/mL streptomycin. Tissues were mechanically dissociated by successive aspirations of the medium and cells counted in a Neubauer Chamber. For immunofluorescence experiments, cells were seeded on coverslips at a density of 3×10^6^ cells/dish (3.1×10^3^ cells/mm^2^). For western blotting experiments, 10^7^ cells were seeded on 35 mm plastic culture dishes (1.04×10^4^ cells/mm^2^). Cells were then incubated at 37°C for the indicated periods of time, in humidified atmosphere of 95% air/5% CO_2_. The culture medium was changed every other day.

### Retinal explants

Explants of chick embryo retinas were prepared as described previously for the rat retina [30]. Briefly, retinas from 7-days-old chick embryos (E7) were dissected in culture medium, cut into fragments of 1 mm^2^ that were placed in 25 mL tight-lidded flasks containing 3 mL of MEM buffered with 20 mM HEPES, pH 7.4, containing 2% fetal calf serum and antibiotics. The flasks were kept in an orbital shaker at 80–90 rpm, at 37°C, for the periods indicated. Drugs were added at least 2–4 h after the explants preparation.

### Labeling of cycling cells with BrdU

To distinguish cycling cells in culture, we used BrdU that is incorporated instead of thymidine by cells synthesizing DNA [31]. Monolayer cultures were incubated with 3 mg/mL of BrdU for 6 h to label proliferating cells, washed and maintained at 37°C for additional 16 h to ensure that labeled cells reached mitosis.

### Fixation and histological processing

After 1–2 days, cultures were fixed with cold 4% paraformaldehyde in 0.16 M sodium phosphate buffer, pH 7.4, for 30 min and extensively washed with phosphate buffered saline (PBS). Intact retina and explanted retinas were fixed by immersion in cold 4% paraformaldehyde in 0.16 M sodium phosphate buffer, pH 7.4, for 1 h, washed and cryo-protected with 30% sucrose in the same buffer. Explants were oriented in an aluminum chamber filled with OCT embedding medium under a dissecting microscope and frozen. Transverse sections (12 µm) were mounted on poly-L-lysine coated glass slides and maintained at 4–8°C until processing.

### Immunofluorescence

For phospho-Akt (p-Akt), phospho-4E-BP1 (p-4E-BP1), phospho-histone H3 (p-H3), γ-tubulin, transitin and cleaved caspase-3, sections or coverslips were permeabilized with PBS −0.25% Triton X-100 for 30 min, incubated with 10 mM citrate buffer, pH 6.0, at 100°C, for 10 min and washed with pure water and PBS. Sections were incubated with a blocking albumin solution (1% BSA in PBS) for 1 h at room temperature and then incubated overnight at 4°C with primary antibodies (p-Akt, 1∶60; p-4E-BP1, 1∶60; transitin, 1∶100; γ-tubulin, 1∶200; p-H3, 1∶300, cleaved caspase-3, 1∶100). Cells or tissues were washed with PBS and incubated with fluorescent secondary Alexa-antibodies (1∶200) for 2 h at room temperature. Chromosomal DNA was stained with DAPI and sections were mounted in phosphate buffer containing 4% n-propylgalate and 80% glycerol. For BRDU detection, cells on coverslips were first permeabilized with PBS-0.25% Triton X-100, incubated with 2N HCl at 37°C for 10 min and washed with 0.1 M Borate Buffer pH 8.5 and PBS before non-specific binding blockade and incubation with the primary antibody (BRDU 1∶500). Some sections were incubated in the absence of the primary antibody and no immunoreactivity was detected under this condition. Imaging was performed with a Leica fluorescence microscope or a Leica SP5 confocal microscope. Image analysis was performed with Adobe Photoshop software.

### Cell treatment and western blotting

Retinal cells obtained from 7-day-old embryos and cultured as monolayers for 1 day (E7C1) were incubated with 10 or 25 µM LY 294002 for 24 h at 37°C and then transferred to sample buffer without bromophenol blue (62.5 mM Tris-HCl, pH 6.8, containing 10% glycerol, 2% SDS and 5% 2-mercaptoethanol). Similarly, retinal explants treated with 25 µM LY 294002 (LY) were transferred to sample buffer and vigorously mixed in Vortex. In other experiments, retinal explants were pre-incubated with LY for 12 or 24 h and transferred to sample buffer at the same time after incubation in order to avoid differences in their developmental stage. Control, untreated cultures or explants were also collected at the end of incubation. Cell extracts were boiled and centrifuged at 27.000×g for 10 min to remove non-soluble material. Protein contents in 2 µL samples of extracts were estimated by the Bradford protein assay [32], using BSA solution containing 2 µL sample buffer as standard. After addition of bromophenol blue (0.02%), extract samples (50 µg/lane) were size fractionated on 12% SDS polyacrylamide gel, transferred to PVDF membranes (GE Healthcare), stained with Ponceau red and blocked in Tris-buffered saline (pH 7.6) with 0.1% Tween-20 and 5% non-fat milk or 5% BSA for Cyclin B1 and CDK1 detection. Membranes were incubated with diluted primary antibody (p-H3, 1∶3000; p-Akt, 1∶4000; p-4E-BP1, 1∶4000; p-GSK3, 1∶8000; Cyclin B1, 1∶1000; CDK1, 1∶2000; PCNA, 1∶4000; p-CDK1, 1∶1500) overnight, at 4°C. Blots were developed using a secondary antiserum conjugated to horseradish peroxidase (Bio-Rad Labs Inc.) and enhanced chemiluminescence, according to the manufacturer's protocol (ECL plus, GE Healthcare). In selected experiments, membranes were stripped and re-probed with anti-Akt (1∶2000), anti-α-tubulin (1∶70000) or anti-ERK (1∶2000), at 4°C, followed by incubation with the secondary antibody and detection as described above. The intensities of labeled bands in western blot experiments were quantified by using Scion Image Software. Comparisons were made by one-way analysis of variance (ANOVA) followed by the Bonferroni post-test.

### TUNEL assays

Retinal explants treated or not with LY 294002 were fixed with 4% paraformaldehyde for 1 h and washed 3 times with PBS. Apoptotic cells were labeled with the APO-BrdU ™ TUNEL Assay Kit (Molecular Probes) according to the provided protocol. Labeled cultures were photographed on a Nikon Eclipse microscope under fluorescence illumination. Experiments were replicated 3 times with similar results.

### Counting of phospho-histone H3^+^, cleaved caspase-3^+^ cells

Transverse sections of control or LY294002-treated retinal explants with 150 µm of linear extent parallel to the retina surface were divided in 3 segments with the same width. The outer, medium and inner segments corresponded to the photoreceptor side, central portion and vitreous side of the retina, respectively. Labeled cells were counted in the 3 segments of 10 different sections of each explant preparation. Four separate experiments were performed. The statistical analysis was performed by ANOVA and the Bonferroni's multiple comparison test.

## Results

**Figure 1:**
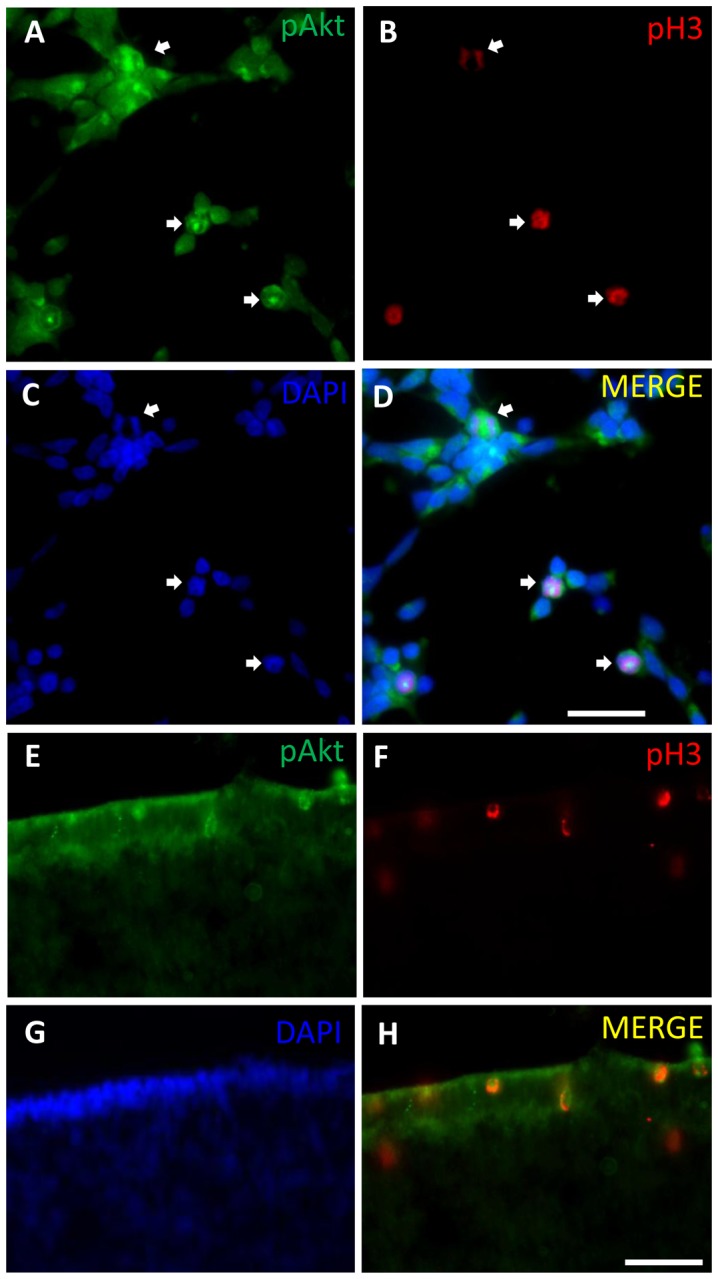
Phospho-Akt expression in mitotic cells in retinal monolayer cultures and intact embryo retina. (A–D) Retinal cell cultures from 7-day-old chick embryos maintained for 1 day (E7C1) were fixed and assayed for immunofluorescence against phospho-Akt (A) and phospho-histone H3 (B). Nuclei were stained with DAPI (C). Merged figures in D. (E–H) Retinal sections from 8-day-old embryo retinas were assayed for immunofluorescence against phospho-Akt (E) and phospho-histone H3 (F). DAPI staining of nuclei (G). Merged figures in (H). Arrows point to double labeled mitotic cells. Scale bar  = 20 µm in A–D and 30 µm in E–H.

PI3K/Akt pathway has been shown to be involved in meiotic maturation in several species [13], [15], [16], [17] and in some cell lines it seems to impair the transition from G2 to M phase of the cell cycle [18], [19]. In order to verify if PI3K pathway was related to mitosis in the retina, the expression of phosphorylated Akt was investigated in retinal cell cultures obtained from 7-day-old embryos and cultured for 1 day (E7C1), a stage where late developing retinal progenitors are still proliferating. Level of phospho-Akt was low in the majority of cells in culture. However, a marked expression of phospho-Akt in mitotic cells could be easily recognized by their phospho-histone H3 (p-H3) labeling and DAPI-labeled condensed chromosomes (Fig. 1A–D). The presence of phospho-Akt in dividing retinal cells expressing p-H3 was also observed in sections of 8-day-old embryo retinas (Fig. 1E-H). Most of these double labeled cells were observed at the ventricular margin of the retina where mitosis of cycling progenitors occurs. No labeled cells were observed in control sections processed without the primary antibody (not shown).

**Figure 2:**
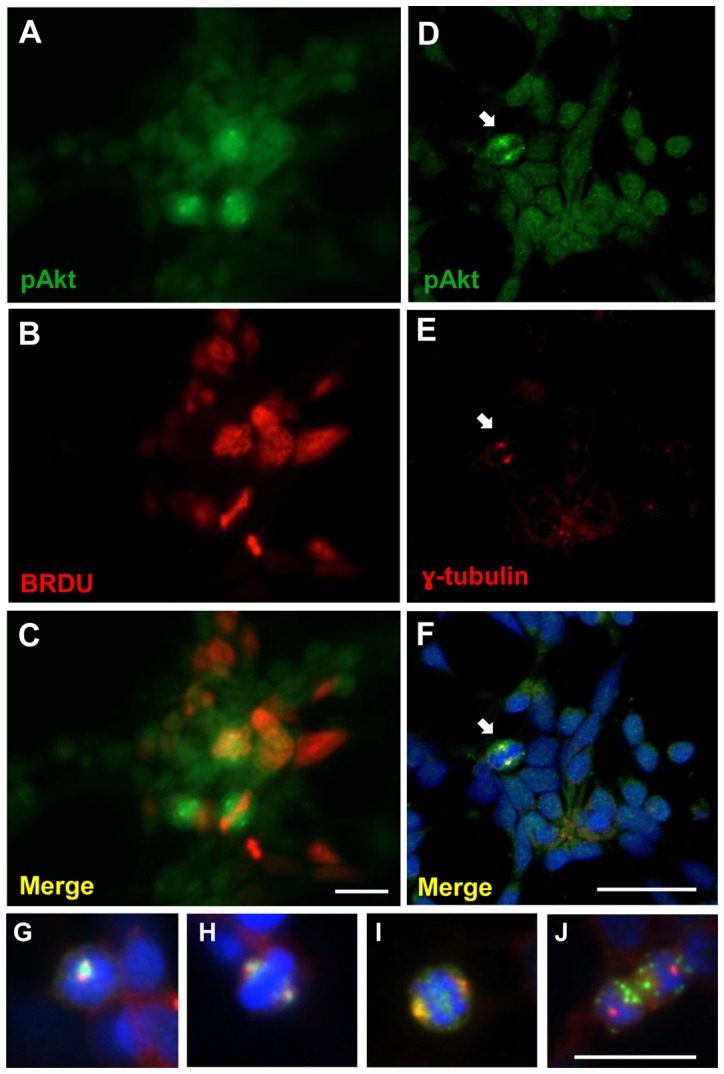
Phospho-Akt is expressed in BrdU positive cells and over centrosomes of dividing cells in retinal monolayer cultures. (A–C) Cultures at E7C1 were incubated with BrdU for 6 h, washed and cultivated for additional 15–16 h in fresh medium. Immunoassays were performed against phospho-Akt (A) and BrdU (B). Merged figures in C. Immunoassays of cultures at E7C1 performed against phospho-Akt (D) and gamma-tubulin (E). Merged figures in F. (G–J) High magnification micrographs showing double labeling against phospho-Akt and gamma-tubulin in dividing cells at prophase (G), metaphase (H), anaphase (I) and telophase (J). Nuclei were stained with DAPI in F–J. Arrow points to double labeled pAkt, γ-tubulin positive cells. Scale bar  = 10 µm in A–C and G, H, I, J and 20 µm in D–F.

To confirm that phospho-Akt staining occurred in retinal progenitors, monolayer cultures were incubated with BrdU for 6 h to label proliferating cells, washed and maintained for an additional period of time (∼16 h) to ensure that labeled cells reached mitosis. Cells were fixed and labeled against phospho-Akt and BrdU. Double-labeled cells could be distinguished and phospho-Akt staining could be noticed over BrdU-labeled cells with condensed chromosomes (Fig. 2A–C). Furthermore, fluorescent phospho-Akt labeling in mitotic cells was more intense at the points corresponding to the centrosomes that were labeled with antiserum against gamma-tubulin (Fig. 2D–F). Phospho-Akt together with gamma-tubulin labeling was clearly noticed over centrosomes of diving cells in prophase (Fig. 2G), metaphase (Fig. 2H) and anaphase that also expressed phospho-Akt at the middle of the mitotic spindle (Fig. 2I). In dividing cells in telophase, no double-labeling was noticed and a punctated phospho-Akt staining was detected over the cell cytoplasm (Fig. 2J).

**Figure 3:**
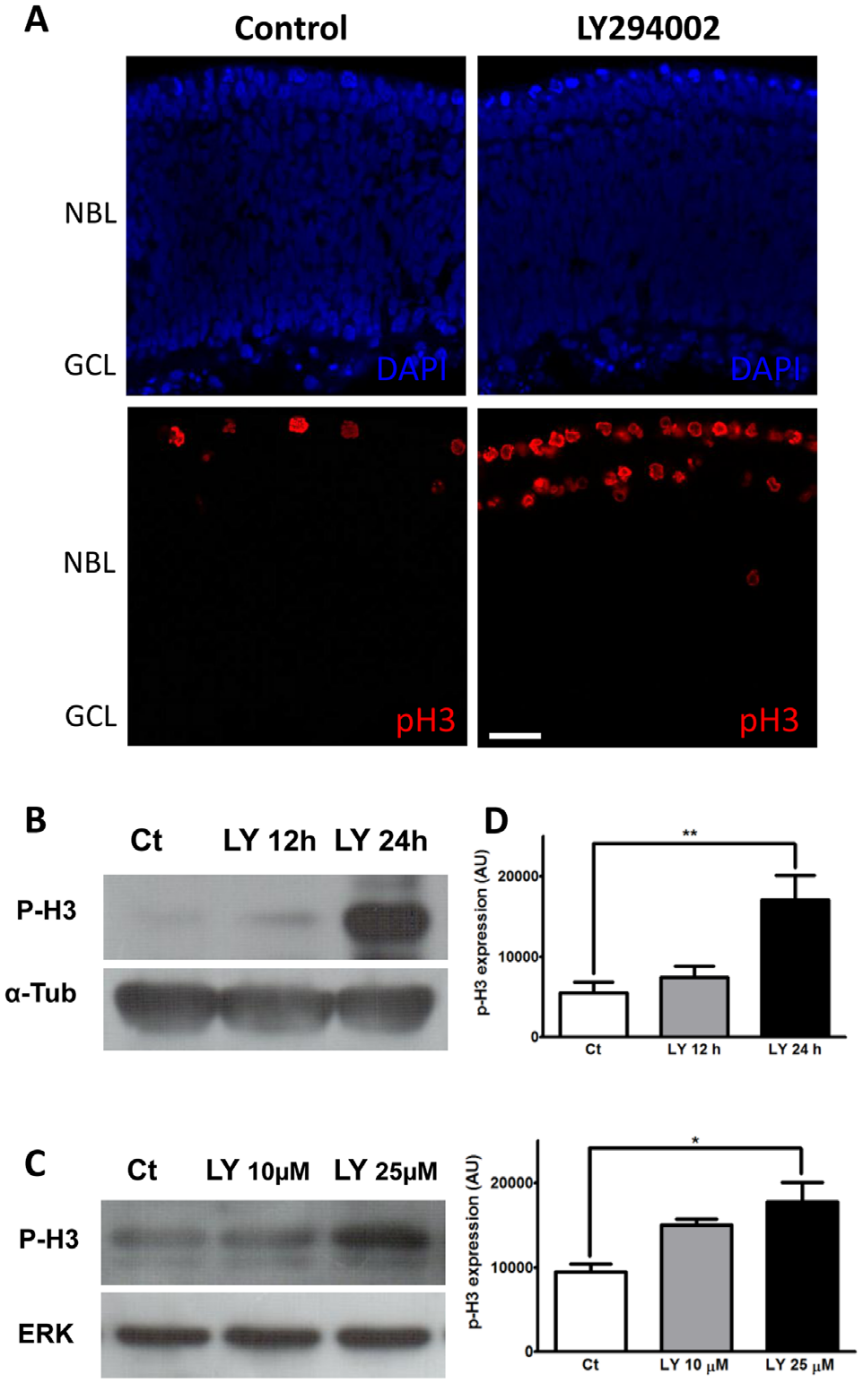
Inhibition of PI3K increases phospho-histone H3 expression in retinal explants and monolayer cultures. (A) Retinal explants from 7-day-old chick embryos were treated for 22 h with 25 µM LY294002, fixed and labeled with DAPI (blue) or antiserum against phospho-histone H3 (P-H3) (red). Control explants were cultured for the same period without inhibitor. Note the increase in the number of p-H3-positive cells at the border of the treated retina. (B) Explants of retinas from embryo at E7 were treated with 25 µM LY 294002 for 12 or 24 h and processed for P-H3 detection by western blot. (C) Retinal monolayer cultures at E7C1 were treated for 24 h with 10 µM or 25 µM LY 294002 and processed for P-H3 detection. Protein gel loading was assessed with anti-α-tubulin and anti-ERK antisera, respectively. Representative blots from at least 3 independent experiments are shown. (D) Quantification of blots represented in B and C. Data represent the mean ± S.E.M. in arbitrary units (A.U.) of 5–6 experiments performed in duplicate or triplicate. **p<0.01 and *p<0.05, compared to control cultures without LY treatment. Ct  =  control cultures or explants cultivated without drugs. NBL  =  Neuroblastic Layer. GCL  =  Prospective Ganglion Cell Layer. Scale bar  = 20 µm.

The pattern of expression of phospho-Akt observed at the diverse stages of mitosis suggests that regulated activation of PI3K/Akt pathway is involved in cell mitosis in the retina. To evaluate this hypothesis, explants of retinas obtained from 7-day-old chick embryos were incubated for 22 h with the PI3K inhibitor LY 294002 and processed for detection of phospho-histone H3 by immunofluorescence, as during cell cycle, expression of this protein phosphorylated at serine 10 is initiated in the pericentromeric heterochromatin of cells at late G2. As mitosis proceeds, p-H3 increases and spreads along chromosomes until prophase. During anaphase, it decreases and is absent during telophase [33], [34], [35]. As can be noticed in Fig. 3A, LY294002 promoted a substantial increase in the number of p-H3 positive cells, most of them confined in the apical, ventricular margin of the NBL of the retinal explants. No significant increase in the number of ectopic labeled cells was detected and a pool of intensively labeled cells, most likely at late G2 or prophase, could be distinguished by their chromatin appearance.

In order to confirm that p-H3 positive cells accumulated with PI3K pharmacological inhibition, retinal explants were treated with 25 µM LY for 12 or 24 h and processed for the detection of p-H3 by western blot (Fig. 3B). A significant increase of ∼310% in the expression of this protein was observed in explants treated with LY for 24 h (Fig. 3D). A similar increase in the expression of p-H3 was observed in monolayer cultures of retinal cells that were cultivated for 24 h in presence of 25 µM LY (Figs. 3C, D).

**Figure 4:**
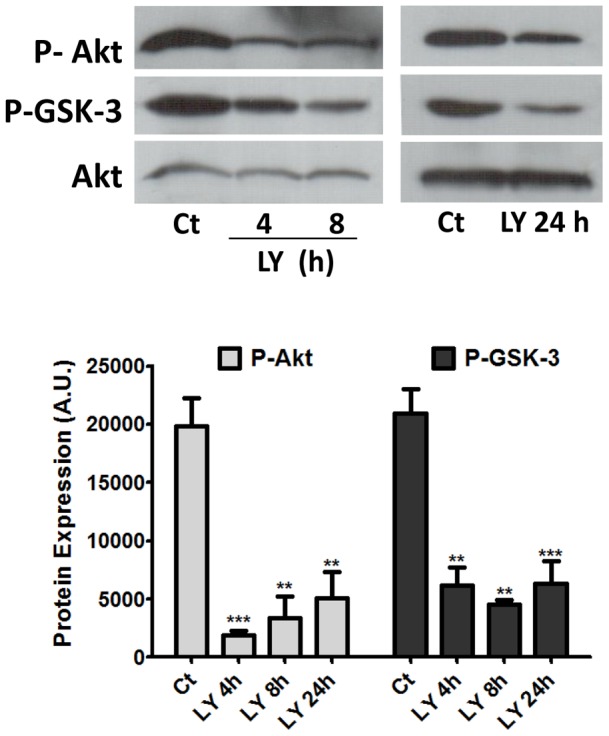
PI3K inhibition decreases phospho-Akt and phospho-GSK-3 levels in retinal cells. Retinal explants from 7-day-old chick embryos were treated with 25 µM LY 294002 for 4, 8 or 24 h and protein extracts processed for detection of phospho-Akt and phospho-GSK3 expression. Gel loading was assessed with anti-Akt antiserum. Blots were quantified by densitometry and data are expressed as the mean ± S.E.M. of arbitrary units. **p<0.01 and ***p<0.001 relative to control. In both cases, representative blots from at least 3 independent experiments are shown. Ct  =  control explants cultivated without drug.

The effect of PI3K inhibition on the phosphorylation of Akt and GSK3 was also evaluated. Retinal explants were treated for several periods of time with LY 294002 and processed for western blot. A substantial decrease in the levels of phosphorylation of both downstream kinases was observed (Fig. 4). Explants maintained for 4 and 8 h in the presence of the inhibitor showed very low levels of phospho-Akt and phospho-GSK3 that decreased to ∼10% and 17% (p-Akt) and to ∼29% and 22% (p-GSK3) of the control, non-treated levels, respectively. A similar low expression of 26% and 30% of the control levels was observed for phospho-Akt and phospho-GSK3, respectively, after 24 h of incubation of the explants with LY 294002.

**Figure 5:**
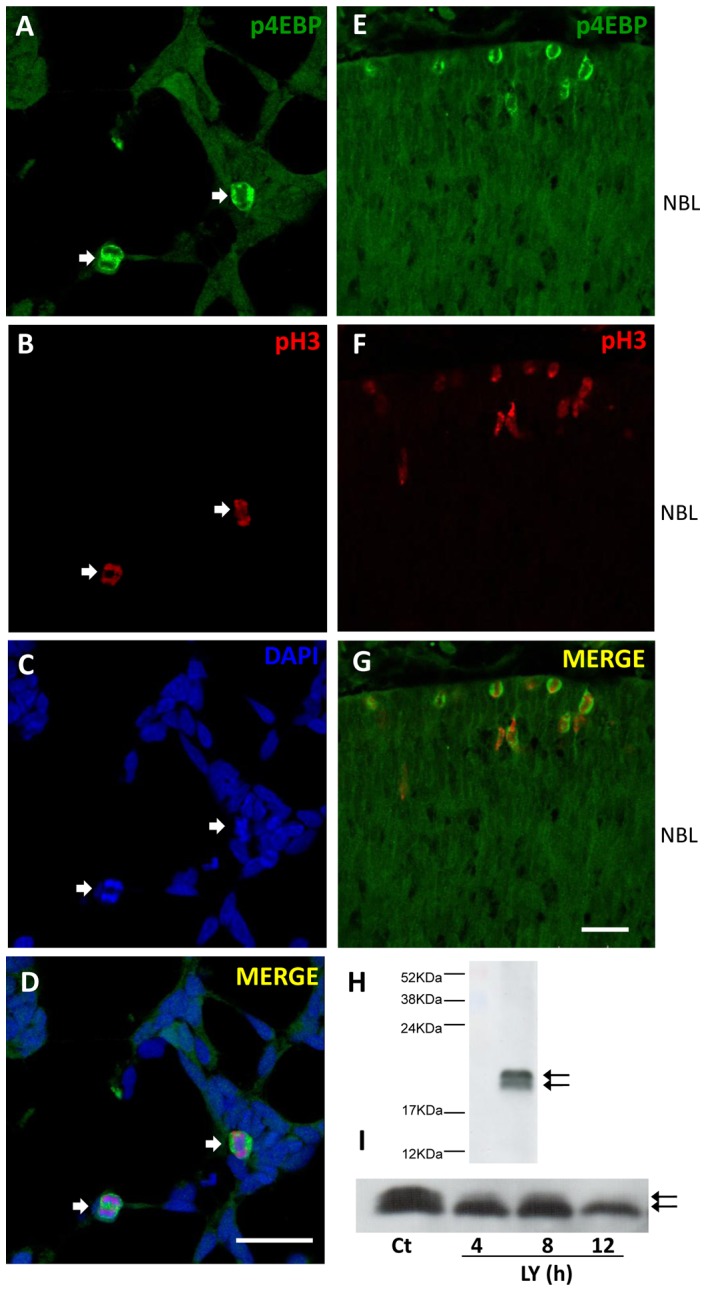
Phospho-4E-BP1 can be easily visualized in mitotic cells of retinal monolayer cultures or intact embryonic retina. (A–D) Retinal cell cultures at E7C1 were fixed and assayed by immunocytochemistry for phospho-4E-BP1 (A) and phospho-histone H3 (B). DNA was stained with DAPI (C) and merged figures are shown in (D). Arrows point to double labeled cells. (E-G) Phospho-4E-BP1 labeling in mitotic cells of 8-day-old chick embryo retinas. Retinal sections were stained with anti-phospho-4E-BP1 (E) and anti-phospho-histone H3 (F). Merged figures are shown in (G). Phospho-4E-BP1 labeled cells were confined to the ventricular margin of the retina. (H) Detection of phospho-4E-BP1 in extracts of retinal cultures at E7C1 without any treatment. The phosphorylated forms γ and β of the protein are indicated by arrows, respectively. (I) Expression of phospho-4E-BP1 in extracts of retinal explants treated with 25 µM LY 294002 for 4, 8 or 12 h. Representative blots from at least 3 independent experiments are shown. Ct  =  control explants cultivated without drug. NBL  =  Neuroblastic Layer. Scale bar  = 20 µm.

Stimulation of cells with growth factors markedly increases the phosphorylation of eIF4E-binding protein (4E-BP1) and promotes its dissociation from the 4E-BP1-eIF4E complex, thus increasing protein synthesis. PI3K/Akt signaling pathway, together with FRAP/mTOR complex, is known to induce the phosphorylation of the 4E-BP1 [36], [37]. Similar to phospho-Akt, 4E-BP1 protein was found to be phosphorylated in cultured retinal cells during mitosis. As can be noted in figure 5A–D, a marked expression of phosphorylated 4E-BP1 was detected in the cytosol of dividing cells in mitosis. No co-localization with DNA evidenced by p-H3 and DAPI staining was observed. Expression of phospho-4E-BP1 in mitotic cells was also observed in the intact retinal tissue (Fig. 5E-G). In sections of 8-day-old embryo retinas, labeling against phosphorylated 4E-BP1 was observed in cells located at the ventricular margin of the NBL that also expressed p-H3.

PI3K/Akt signaling can affect the translation of mRNAs through the phosphorylation of downstream targets such as 4E-BP proteins and S6 kinase [37], [38]. Under denaturing conditions, highly phosphorylated γ form of 4E-BP1 protein migrates on SDS-polyacrylamide gels more slowly than low β or non-phosphorylated α forms. In order to verify if the phosphorylation state of 4E-BP1 protein was affected by PI3K inhibition, extracts of chick retinal cultures at E7C1 were probed with antibodies against phospho-4E-BP1. Two close but discernible gel bands were noticed, likely corresponding to the hyper and hypo-phosphorylated forms of the protein (Fig. 5H). A clear decrease in the expression of the hyper-phosphorylated form of 4E-BP1 was detected when cultures were treated with 25 µM LY294002 for increasing periods of time (Fig. 5I).

**Figure 6:**
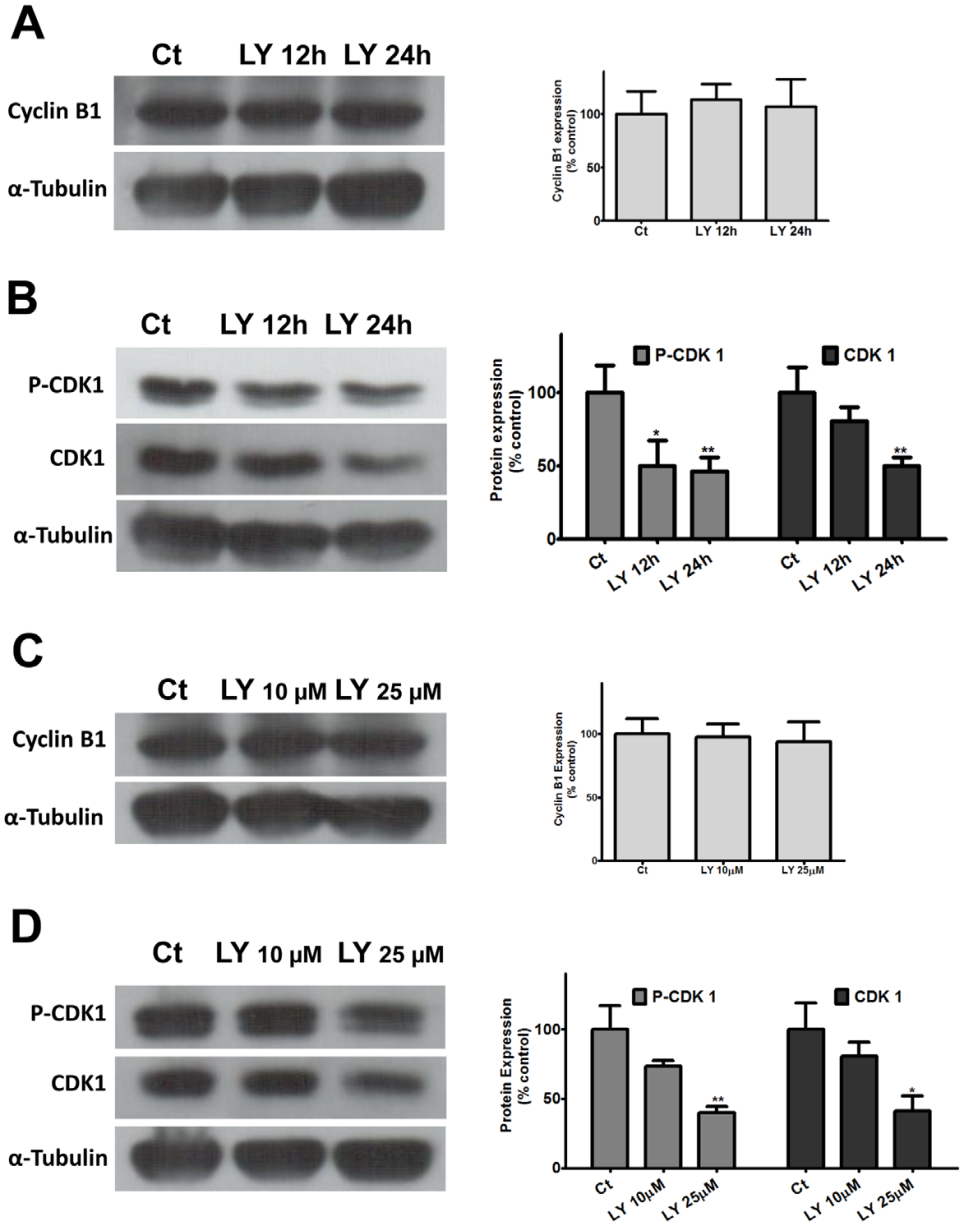
Inhibition of PI3K/Akt pathway modulates expression of cell cycle proteins in retinal explants (A, B) and monolayer cultures (C, D). Retinal explants from 7-day-old chick embryos (A, B) were treated with 25 µM LY 294002 for 12 or 24 h and processed for the detection of cyclin B1 (A), phospho-CDK1 and CDK 1 (B). Monolayer cultures at E7C1 were treated with 10 µM or 25 µM LY 294002 for 24 h and processed for detection of cyclin B1 (C), phospho-CDK 1 and CDK1 (D). Representative blots from at least 3 independent experiments are shown. Gel loading was assessed with anti-α-tubulin antiserum. Blots were quantified by densitometry and data represent the mean ± S.E.M. (% control). *p<0.05 and **p<0.01 relative to control.

In most animal cells, mitosis is triggered by activation of the cyclin-dependent kinase CDK1, also known as cdc2. Activation of this kinase is a multi-step process that begins with the binding of cyclin B whose level rises at G2 and peaks in mitosis [39]. In order to investigate if blockade of G2/M transition induced by inhibition of PI3K/Akt pathway was due to the modulation of some of these regulatory proteins, we first evaluated the expression levels of cyclin B1 in retinal explants treated with LY294002 (Fig. 6A). No significant change in the expression of cyclin B1 was detected when explants were treated with 25 µM of the inhibitor for 12 or 24 h.

Phosphorylation of CDK1 at the Tyr15 residue was also shown to be involved in the arrest of dividing cells at G2/M transition [40]. A significant decrease in the levels of phosphorylated CDK1 was observed when explants were incubated with 25 µM LY (Fig. 6B). Phospho-CDK1 levels decreased to 50% and 46% of the control levels after 12 h and 24 h of incubation, respectively. Levels of non-phosphorylated CDK1 also decreased significantly to 50% of the control, non-treated culture levels, after incubating retinal explants with LY294002 for 24 h. A small decrease of 19% in CDK1 expression levels could already be noticed after a 12 h incubation of the explants with LY, although the effect of the inhibitor did not reach statistical significance at this time point.

Similar to retinal explants, no significant change in cyclin B1 levels could be noticed when retinal monolayer cultures at E7C1 were incubated for 24 h with 10 µM or 25 µM LY294002 (Fig. 6C). In contrast, inhibition of PI3K/Akt pathway with 25 µM LY 294002 led to a reduction of ∼60% in the levels of phospho-CDK1 and total CDK1, as compared to control, non-treated cultures (Fig. 6D).

**Figure 7:**
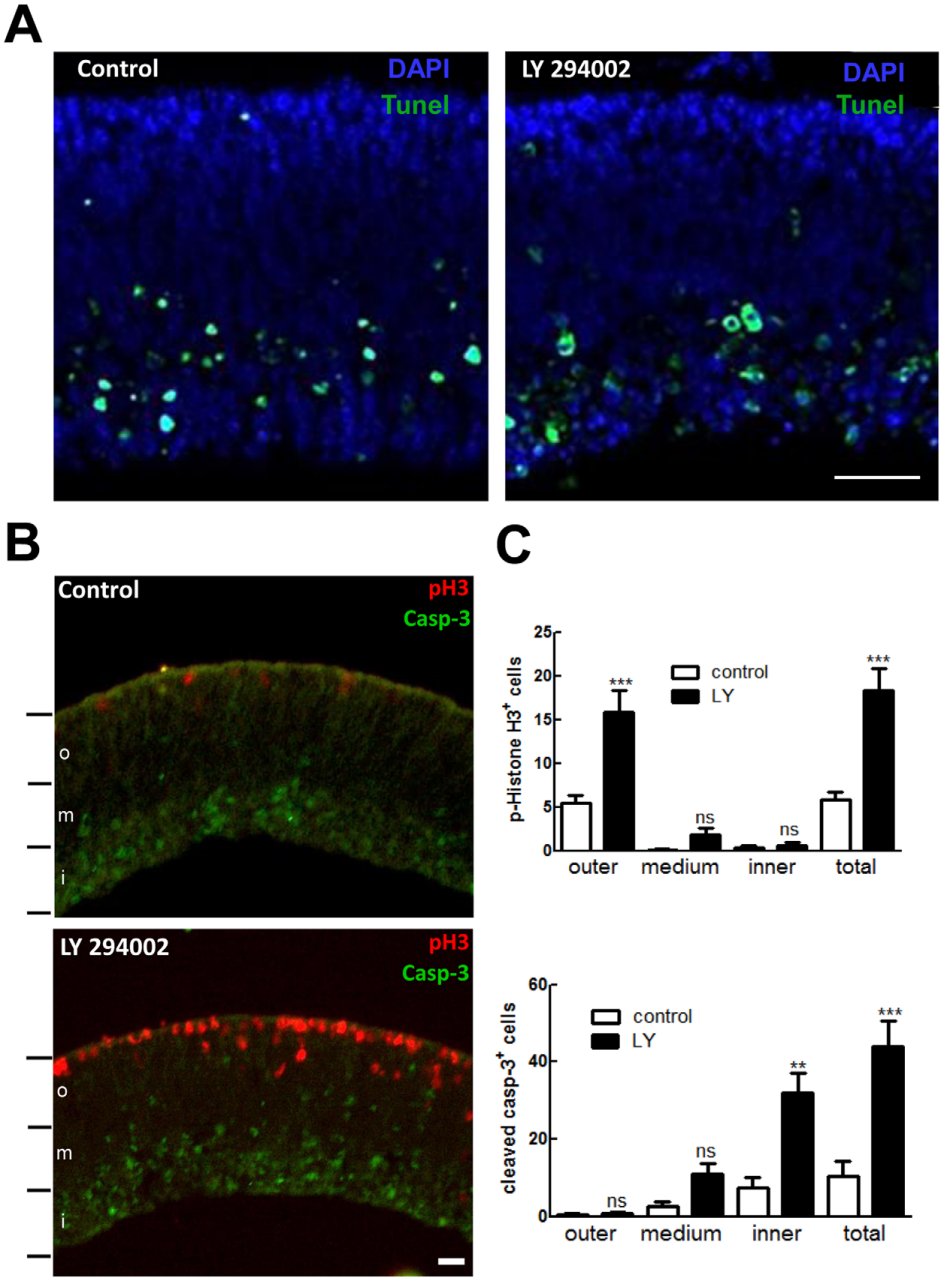
Inhibition of PI3K with LY 294002 does not alter the pattern of apoptotic cells in the outer retina. (A) Retinal explants from 7-day-old chick embryos were treated for 22 h with 25 µM LY 294002, fixed and sections processed by TUNEL assays as described in methods. Representative micrographs of TUNEL labeling (green) are shown. DAPI (blue) was used to label nuclei of all cells and explants were oriented with their outer, ventricular margin at the upper side of the micrographs. Data are representative of three experiments. (B) Retinal explants were treated for 22 h with 25 µM LY 294002, fixed and sections processed for immunocytochemistry against cleaved caspase-3 (green) and phospho-histone H3 (red). (C) Quantification of phospho-histone H3 and cleaved caspase 3 positive cells in retinal explants. Positive cells were counted in 10 transverse sections of retinal explants with 150 µm of linear extent parallel to their surface that were divided in 3 segments with the same width (o  =  outer segment; m  =  medium segment; I  =  inner segment). Data represent the mean ± S.E.M. of cell counts/segment from four independent experiments. Scale bar  = 20 µm.

PI3K/Akt pathway is involved in the survival of several cell types, including neurons of the mouse retina [39]. In order to verify if inhibition of PI3K was causing the death of dividing retinal progenitors, retinal explants at E7C1 were treated with 25 µM LY294002 for 22 h, fixed and processed for TUNEL or immunocytochemistry against cleaved caspase-3 and phospho-histone H3 to estimate the localization and amount of apoptotic cells. Both under control or LY294002-treated conditions, apoptotic cells were not consistently found in the ventricular tier of cells where dividing progenitors are located in the retinal explants (Fig. 7). TUNEL positive cells were observed only at the inner portion of the retina where presumptive ganglion and amacrine cells are located, both in control and LY 294002-treated explants (Figure 7A). Moreover, in both conditions, cleaved caspase-3 positive cells were mostly found at the inner portion of the retina, where phospho-H3^+^ cells were not observed (Figure 7B). Cells labeled for both markers were not detected in control or in LY 294002-treated explants.

Quantification of cleaved caspase-3 and phospho-histone H3 positive cells in the retinal explants revealed that treatment with LY 294002 induced an increase in both populations of labeled cells (Figure 7C). While the number of phospho-H3^+^ cells/section increased from 5.8±0.9 in control explants to 18.3±2.5 in LY 294002-treated explants, the number of cleaved caspase-3 positive cells/section also increased from 10.4±4.0 in control tissues to 43.9±6.6 in treated explants. Moreover, in order to further characterize the effect of PI3K inhibition on cell death in the retinal explants, cleaved caspase-3 and phospho-H3 positive cells were counted in three segments of the explants, the outer, the medium and the inner segments corresponding to the photoreceptor side, central portion and vitreous side of the retina, respectively. Quantification of labeled cells in the three segments revealed that while LY 294002 significantly increased the number of phospho-H3^+^ cells only at the outer segment of the explants (in cells/segment: control  = 5.4±0.8; LY 294002 = 15.9±2.5), this compound significantly increased the number of cleaved caspase-3^+^ cells only at the inner segment of the explants (in cells/segment: control  = 7.3±2.8; LY 294002 = 32.1±5.0).

**Figure 8:**
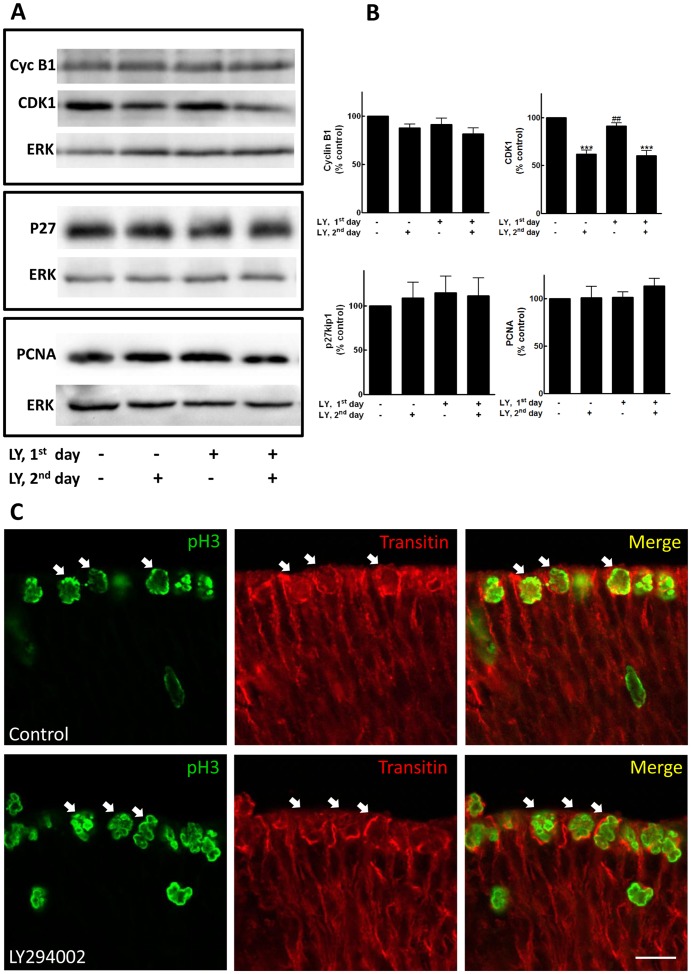
Effect of LY294002 removal on the expression of cell cycle regulatory proteins and development markers. (A) Monolayer cultures of retinal cells from E7 were established as described. After 2 h, LY294002 to a final concentration of 25 µM was added. After 24 h, medium was removed, fresh medium was added and cells cultivated for an additional 24 h period. At the end of incubations, protein extracts were analyzed for the indicated proteins by western blotting. Representative blots are shown. (B) Blots were quantified by densitometry and data are expressed as the mean ± S.E.M. (% control) of 3 or 4 experiments. ***p<0.001 relative to control without treatment. ^##^p<0.01 relative to cultures treated on the second day or during the entire period. (C) Expression of transitin in treated explants. Retinal explants from 7-day-old chick embryos were treated for 24 h with 25 µM LY294002, fixed and labeled with anti-pH3 (green) or antiserum against transitin (red). Arrows point to double labeled cells. Control explants were cultured for the same period without inhibitor. Scale bar  = 10 µm.

Since LY294002 decreased CDK1 levels, PI3K inhibition could be inducing the exit of retinal progenitors from the cell cycle and their differentiation. In order to explore this point, we investigated if the effect of LY294002 on the expression of cell cycle regulatory proteins was reversible. We also investigated the effect of LY294002 on the expression of the cyclin/CDK inhibitor p27kip1 and of the proliferation marker PCNA. Retinal cells at E7 were incubated with 25 µM LY294002 in the first day of culture and then cultivated in fresh medium for an additional period of 24 h (Fig. 8). While LY294002 decreased CDK1 levels when cells were incubated in the last 24 h or during the entire period studied (62±4% and 60±5% of the control levels, respectively), no significant decrease was observed when the inhibitor was added in the beginning of the cultures and removed after 24h (91±3% of the control levels). Moreover, no significant changes in the expression of cyclin B1, p27kip1 and PCNA were observed when cultures were incubated with LY for the different protocols of incubation.

Transitin in an avian homologue of nestin that is highly expressed in retinal progenitors and early developing glial cells [41]. In order to verify if mitotic retinal progenitors expressed transitin after PI3K inhibition, retinal explants were incubated for 24 h with 25 µM LY294002. Then, sections of explants were incubated with anti-pH3 and anti-transitin. No clear difference in the pattern of transitin labeling was detected in explants treated with LY and labeling for transitin was clearly observed in pH3 positive cells located at the ventricular margin of the retina in both control and LY-treated explants (Fig. 8B).

## Discussion

In the present work, we showed that Akt was phosphorylated in mitotic cells, as high labeling for phospho-Akt was observed over BrdU positive cells with condensed chromosomes and over cells labeled for phospho-histone H3 in both monolayer cultures and intact tissues. Although low levels of phospho-Akt could be detected in the majority of cultured cells, high labeling for phospho-Akt was visualized in gamma-tubulin positive centrosomes of mitotic cells in prophase, metaphase and anaphase, suggesting that this enzyme might be active during mitosis of retinal progenitors. In good agreement with this idea are previous evidences showing that in synchronized HeLa, MDCK and NIH3T3 cell cultures an increase in phosphorylation and kinase activity of Akt occurs in S, G2 and early phases of mitosis [18], [19], [42]. Since, besides functioning as microtubule organizing centers, centrosomes are thought to function as scaffolds, bringing together proteins such as cyclin B1, Polo-like kinase-1 (Plk1), Aurora A and cdc25 [39], our findings also suggest that Akt may interact and modulate the activity of proteins located at centrosomes in retinal progenitors in mitosis. Both GSK3 and Wee1, two phosphorylation targets of Akt [43], [44] as well as phospho-Akt itself were also shown to be located at centrosomes of dividing cells [42], [45].

Treatment of retinal cultures with LY294002, besides decreasing significantly phospho-Akt and phospho-GSK3 levels, promoted an increase in the number of phospho-histone H3 positive cells and protein levels that was time and dose-dependent. Bright fluorescence was observed over cells located at or near the ventricular edge of the retina with condensed chromatin similar to cells in late G2 or in prophase. Several possibilities could account for these observations. The first would be that pharmacological inhibition of PI3K/Akt pathway increased the proliferation of retinal progenitors due to an accelerated cell cycle progression that would increase the number of pH3^+^ cells in mitosis after 24 h of treatment. Alternatively, inhibition of PI3K could induce the opposite effect on the cell cycle, that is, a slower mitotic cycle with more pH3^+^ cells in mitosis being visualized after the treatment with LY. In good agreement with this later possibility are previous evidences showing that PI3K inhibition decreases the incorporation of [^3^H]-thymidine and cyclin D1 expression induced by nucleotides in chick embryo retinal cultures [28], suggesting that blockade of PI3K/Akt pathway has an inhibitory effect on the proliferation of late developing retinal progenitors in culture. A similar inhibitory effect of PI3K inhibitors on cell proliferation was observed in several primary and transformed cell types [46].

During development of the quail retina, down-regulation of CDK1 and cyclins A and B2 is coincident with the down-regulation of the proliferation marker PCNA as well as with the emergence of the microtubule-associated protein TAU, a differentiation marker. This coincidence was associated with the exit of progenitors from the cell cycle and neuronal differentiation in this tissue [47]. Here we showed that the increased number of phospho-histone H3 positive cells induced by PI3K inhibition was concomitant with a decrease in the expression of CDK1, the enzyme that triggers the irreversible entry of dividing cells into mitosis when fully activated [10]. Since PI3K inhibition also decreases the incorporation of [^3^H]-thymidine in our cultures [28], the present results raise the possibility that inhibition of PI3K induced the proliferation of retinal progenitors associated with their exit from the cell cycle and differentiation. Although this hypothesis is exciting, no decrease in the expression of cyclin B1 was observed when retinal monolayer cultures or retinal explants were incubated with LY294002 for 24 or 48 h, suggesting that this compound did not induce the down-regulation of cyclin B1 in our preparations. Moreover, the decrease in the expression CDK1 induced by PI3K inhibition was reversible and its level of expression increased to control levels when the inhibitor was removed and the cultures were incubated in fresh medium for an additional period of 24 h. This observation suggests that CDK1 most likely was not down-regulated permanently when retinal cultures were treated with LY294002 and that progenitors did not exit cell cycle upon PI3K inhibition. Reinforcing this idea were the results showing that neither a significant increase in the expression of p27kip1, a cyclin/CDK inhibitor that is expressed in cells that are exiting cell cycle and differentiating in the early developing chick embryo retina [48] nor a decrease in the expression of PCNA, a retinal progenitor marker expressed in all cell cycle phases in the developing mouse retina [49] were observed in the LY-treated cultures. Moreover, the fact that pH3-labeled dividing progenitors from treated cultures expressed transitin, the avian homologue of nestin that is highly expressed in retinal progenitors and early developing glial cells, but not in differentiated retinal neurons or Müller glia [41] also supports this idea.

Another possibility to explain the decreased levels of CDK1 enzyme in retinal preparations treated with LY294002 would be that PI3K inhibition reduced the viability of diving progenitors that would result in a lower number of mitotic cells expressing this enzyme after treatment. A recent work has shown that PI3K pharmacological inhibition induces the mitotic death of HeLa cells [50]. Indeed, treatment of retinal explants with the inhibitor induced an increase in TUNEL and cleaved caspase-3 positive cells in the retinal explants, suggesting an increase in cell death induced by PI3K inhibition. However, the distribution of mitotic phospho-histone H3^+^ cells and cleaved caspase-3^+^ cells was quite different along the thickness of the treated explants. While the amount of phospho-H3^+^ cells significantly increased only at the ventricular region of the treated retina where mitosis is expected to occur, the number of cleaved caspase-3^+^ cells increased only at the inner segments of the treated tissue where presumptive ganglion and amacrine cells are located. Since no phospho-H3^+^, cleaved caspase-3^+^ double labeled cells were observed, the present data suggest that, although treatment of the explants with LY294002 increased cell death in the tissue, dying cells mostly likely were not progenitors in mitosis and that the decreased levels of CDK1 in LY 294002-treated explants were not due to the death of these cells. In good agreement with this idea are our findings showing that no decrease in cyclin B1 and PCNA levels was detected in the treated explants or monolayer cultures and that the levels of CDK1 could be restored after the removal of the inhibitor from the cultures.

Pharmacological inhibition of PI3K with LY294002 affects the G1/S passage of progenitors in retinal monolayer cultures as this compound prevents the increase in [^3^H]-thymidine incorporation and cyclin D1 expression induced by nucleotides [28]. Thus, as opposed to the possibilities discussed before, our findings showing an increase in the number of pH3^+^ cells and in pH3 protein levels could reflect an arrest or delay of progenitors in the G2/M transition in the retinal preparations treated with the PI3K inhibitor. In cell lines such as NIH 3T3 and HeLa cells, transcription of CDK1 mRNA and CDK1 protein synthesis and degradation are tightly coupled to the entry of cells into S phase in every round of the cell cycle and synchronized cultures show a significant increase in CDK1 turnover in the beginning of the S phase of the cell cycle [51], [52]. These evidences together with the present findings suggest that an impairment of G2/M transition in the retinal cultures and explants treated with LY294002 might be due to the blockade in the passage of retinal progenitors from G1 to S phase of the cell cycle rather than to a direct effect of the inhibitor on the G2/M transition. In this scenario, the decrease in CDK1 levels induced by the PI3K inhibitor during the S phase of the cell cycle would delay the formation of the threshold concentration of the cyclin B1-CDK1 complexes necessary for the entry of progenitors into mitosis and an increase in the number of pH3+ cells arrested at the ventricular region of the retina would be observed in LY294002 treated retinal explants. To distinguish if activation of PI3K/Akt pathway induces CDK1 gene transcription and/or CDK1 protein synthesis in proliferating retinal progenitors is an interesting point to be further explored.

Transcription of cyclin B1 mRNA and cyclin B1 synthesis are also increased during S and G2 phases in cell lines [53] and a decrease in the expression of cyclin B1 would be expected to occur if LY294002 treatment blocked retinal progenitors in the G1/S transition. Here, in contrast to CDK1 levels that decreased upon PI3K inhibition, no decrease in the levels of cyclin B1 was detected when retinal cultures were incubated for 24 or 48 h with LY294002 or when the inhibitor was removed from the cultures after a treatment of 24 h. Since cyclin B1 is only down-regulated during mitosis and in the beginning of G1 [53], our data suggest that inhibition of PI3K/Akt pathway did not affect the synthesis and/or stability of cyclin B1 in dividing retinal progenitors that have entered into S phase of the cell cycle before PI3K inhibition. In good agreement with this hypothesis is a recent work showing that PI3K inhibition induces the arrest of glioblastoma cells in G2/M transition by down-regulating CDK1 and cdc25 expression without affecting the expression of cyclin B1 [54].

Finally, it is well known that hyperphosphorylation of 4E-BP1 is required to disrupt its interaction with the eIF4E translation initiation factor [55], [56] and activate cap-dependent translation of RNAs. Although classical cap-dependent translation of RNAs and protein synthesis are impaired during mitosis, translation of specific mRNA by an alternative cap-independent mechanism mediated by Internal Ribosome Entry Sites (IRES) was observed during G2 and mitosis in HeLa and Leukemia cell lines [57], [58]. As mentioned before, phosphorylated Akt was highly expressed in dividing retinal progenitors and inhibition of PI3K with LY294002 induced a decrease in the levels of phospho-Akt, phospho-GSK3 as well as the hyperphosphorylated form of 4E-BP1 in extracts of retinal explants. Since high levels of phospho-4E-BP1 were detected in pH3 positive retinal cells both in primary cultures and intact developing tissues, our findings showing that PI3K inhibition decreased 4E-BP1 phosphorylation raises the possibility that PI3K/Akt pathway is involved in the regulation of 4E-BP/eIF4E interaction and translation of mRNAs in dividing retinal progenitors during mitosis. Similar hyperphosphorylation of 4E-BP1 was observed in synchronized cell lines during mitosis [59], [60] as well as inhibition of IRES-mediated translation of mRNAs by PI3K inhibitors [58]. If PI3K/Akt pathway is involved in the translation of specific mRNAs during mitosis is an interesting point to be explored.
